# Supplementary material for: Shared decision-making for children with medical complexity in community health services: a scoping review
Source: BMJ Paediatr Open. 2023 Apr 3;7(1):e001866. doi: 10.1136/bmjpo-2023-001866 (PMC10083859; doi:10.1136/bmjpo-2023-001866)
Supplement: Author's manuscript [file bmjpo-2023-001866.draft_revisions.pdf]

BMJ Paediatrics Open

BMJ Paediatrics Open is committed to open peer review. As part of this commitment we make the peer review history of every article we publish publicly available.

When an article is published we post the peer reviewers' comments and the authors' responses online. We also post the versions of the paper that were used during peer review. These are the versions that the peer review comments apply to.

The versions of the paper that follow are the versions that were submitted during the peer review process. They are not the versions of record or the final published versions. They should not be cited or distributed as the published version of this manuscript.

BMJ Paediatrics Open is an open access journal and the full, final, typeset and author-corrected version of record of the manuscript is available on our site with no access controls, subscription charges or pay-per-view fees (http://bmjpaedsopen.bmj.com).

If you have any questions on BMJ Paediatrics Open's open peer review process please email info.bmjpo@bmj.com

BMJ Paediatrics Open

Shared decision-making for children with medical complexity in community health services – a scoping review

Journal:	*BMJ Paediatrics Open*
Manuscript ID	bmjpo-2023-001866
Article Type:	Original research
Date Submitted by the Author:	16-Jan-2023
Complete List of Authors:	Jacobs, Sonja; Barts Health NHS Trust<br>Davies, Nathan; University College London, Research Department of Primary Care & Population Health<br>Butterick, Katherine L; Barts Health NHS Trust<br>Oswell, Jane L; Barts Health NHS Trust<br>Siapka, Konstantina; Barts Health NHS Trust<br>Smith, Christina; University College London
Keywords:	Health services research

SCHOLARONE™
Manuscripts

BMJ

I, the Submitting Author has the right to grant and does grant on behalf of all authors of the Work (as defined in the below author licence), an exclusive licence and/or a non-exclusive licence for contributions from authors who are: i) UK Crown employees; ii) where BMJ has agreed a CC-BY licence shall apply, and/or iii) in accordance with the terms applicable for US Federal Government officers or employees acting as part of their official duties; on a worldwide, perpetual, irrevocable, royalty-free basis to BMJ Publishing Group Ltd ("BMJ") its licensees and where the relevant Journal is co-owned by BMJ to the co-owners of the Journal, to publish the Work in this journal and any other BMJ products and to exploit all rights, as set out in our licence.

The Submitting Author accepts and understands that any supply made under these terms is made by BMJ to the Submitting Author unless you are acting as an employee on behalf of your employer or a postgraduate student of an affiliated institution which is paying any applicable article publishing charge ("APC") for Open Access articles. Where the Submitting Author wishes to make the Work available on an Open Access basis (and intends to pay the relevant APC), the terms of reuse of such Open Access shall be governed by a Creative Commons licence – details of these licences and which Creative Commons licence will apply to this Work are set out in our licence referred to above.

Other than as permitted in any relevant BMJ Author's Self Archiving Policies, I confirm this Work has not been accepted for publication elsewhere, is not being considered for publication elsewhere and does not duplicate material already published. I confirm all authors consent to publication of this Work and authorise the granting of this licence.

Confidential: For Review Only

Shared decision-making for children with medical complexity in community health services – a scoping review

Sonja Jacobs[1], Nathan Davies[2], Katherine L Butterick[1], Jane L Oswell[1], Konstantina Siapka[1], Christina H Smith[3]

1, Barts Health NHS Trust, London, UK
2. Research Department of Primary Care & Population Health, University College London, UK
3 Division of Psychology and Language Sciences, University College London, UK

Corresponding author: Sonja Jacobs, Community Children's Therapies, 1st Floor, Hastings Ward, Mile End Hospital, Bancroft Road, London, E1 4DG; Sonja.jacobs@nhs.net

Word count: 2499

Keywords:
MeSH headings:

- Decision making, shared
- Professional-Family Relations
- Disabled Children
- Community Health Services

Confidential: For Review Only

ABSTRACT

Background: Children with medical complexity is an increasing population whose parents and healthcare providers face multiple decisions. Shared decision-making is a process where patients, their families and healthcare providers collaborate to make decisions based on clinical evidence and informed preferences of the family. Shared decision-making has benefits for the child, family and healthcare providers, including improved parental understanding of the child's difficulties, increased participation, improved coping skills and more efficient healthcare use. It is however poorly implemented.

Aims and methods: A scoping review was conducted to explore shared decision-making for children with medical complexity in community health services, including how shared decision-making is defined in research, how it is implemented, including barriers and facilitators and recommendations for research. Six databases were systematically searched: Medline, CINAHL, EMBASE, PsycINFO, PubMed, Cochrane Database of Systematic Reviews, and sources of grey literature. The review is reported according to the Preferred Reporting Items for Scoping Reviews.

Results: Thirty sources met the inclusion criteria. Most factors can either be a facilitator or barrier to shared decision-making depending on the context. Two significant barriers to shared decision-making in this population include uncertainty about the child's diagnosis, prognosis, and treatment options and the presence of hierarchy and power-imbalance during clinical encounters with healthcare providers. Further influencing factors include continuity of care, the availability of accurate, accessible, adequate, and balanced information and the interpersonal and communication skills of parents and healthcare providers.

Conclusion: Uncertainty about diagnosis, prognosis, and treatment outcomes for children with medical complexity are additional challenges to the known barriers and facilitators to shared decision-making in community health services. Effective implementation of shared decision-making requires advancement of the evidence-base for children with medical complexity, reducing power-imbalance in clinical encounters, improving continuity of care, and improving the availability and accessibility of information resources.

What is already known on this topic

Shared decision-making is an evidence-based approach with known benefits to children with medical complexity, their families, and the healthcare system, it is however poorly implemented.

What this study adds

This study highlights the impact of uncertainty of diagnosis, prognosis, and treatment outcomes for children with medical complexity on shared decision-making. It highlights how healthcare providers can improve the implementation of shared decision-making by addressing the power-imbalance in clinical encounters, improving continuity of care, improving communication and interpersonal skills, and making information more accessible to parents from diverse backgrounds.

How this study might affect research, practice, or policy

This review can guide a research strategy in the field of shared decision-making for children with medical complexity in community health services support healthcare professionals to consider their influence on the decision-making process in everyday practice.

Confidential: For Review Only

INTRODUCTION

Children with medical complexity (CMC) have needs in four domains: 1) substantial family-identified healthcare and special educational needs, 2) one or more severe and potentially lifelong chronic conditions, 3) limitations to body structure and function, performance of activities and participation that may require technological assistance such as feeding tubes, and 4) high projected healthcare use including the involvement of multiple subspecialties [1]. These children are increasing in number [1,2]. Parents and healthcare providers (HCPs) for CMC face multiple, complex decisions throughout their childhood including decisions about tube feeding, mechanical ventilation, medications, and surgery [1,3,4].

Shared decision-making (SDM) is an evidence-based approach that is an essential part of patient-centred care [5–7]. It is a process where parents, as surrogate decision-makers for their child, and HCPs work in partnership to make decisions based on clinical evidence and family preferences [7–10]. This approach is supported by policy makers and regulatory bodies nationally and internationally [5–10]. The benefits for patients, families, and HCPs include improved patient or carer knowledge and understanding, reduced decisional conflict, increased participation and engagement in care, improved coping skills, and efficient use of healthcare resources [5,6,8,10]. SDM is however poorly defined due to the interpretive nature of what is meant by "shared" [6,11], with fundamental differences in how patients, carers and HCPs understand the purpose of and their role in SDM [6,8,12–14].

The difference between parental and HCP approaches to decision making often result in poor implementation of SDM. HCPs base their decisions on clinical and empirical evidence [8] which is often lacking for CMC [1,15]. Parents consider the social, emotional, and psychological impact of decisions on their child, their family and cultural and religious beliefs in addition to potential clinical outcomes [8,11,13]. Parental decisions about what is 'good enough' for their child with medical complexity are often more intensely scrutinised by HCPs than for non-medically complex children, with a lack of awareness or importance given to the impact of decisions on the family [8,11,13–16]. Clinical uncertainty combined with complex family dynamics require HCPs to swap traditional hierarchical and paternalistic approaches to decision-making, where decisions are made based on clinical information and empirical evidence [5,8,11,12,15,17], for an approach that allows parental collaboration and discretion in decision-making [11,15].

The personal and healthcare cost of poor implementation of SDM is amplified in the CMC population due to their significant healthcare use. Understanding factors impacting SDM for CMC will help to improve medical and developmental outcomes, quality of life of children and families and effective use of healthcare resources [5,8,10].

This scoping review aimed to explore the landscape of shared decision-making for children with medical complexity in community health services.

The objectives for this review were to:

1) Explore how SDM is defined in research.

2) Understand to what extent SDM is implemented for CMC in community health services.

3) Consider the differences in SDM between ethnic groups.

4) Identify the barriers and facilitators to SDM for CMC.

5) Provide recommendations for future research.

Confidential: For Review Only

METHODS

A scoping review was conducted following the Joanna Brigs Institute (JBI) manual for evidence synthesis [18–20] and was reported according to the Preferred Reporting Items for Systematic Reviews and Meta-Analyses extension for scoping reviews (PRISMA-ScR) [21].

Protocol and registration

A scoping review protocol was registered on Open Science Framework on 19-05-2022 [16].

Eligibility criteria

Eligibility criteria are outlined in Table 1. SDM included any process involving parents or caregivers in medical decision-making with HCPs [10] and included family-centred practices. Sources reporting on multiple populations or settings were included if results were reported separately or if at least 50% of the results related to the eligible population or setting. Primary research using any methodology, secondary research including systematic reviews, literature, and scoping reviews and editorial or opinion pieces were included.

Table 1 Eligibility criteria

	Included	Excluded
**Population**	Parents and/or caregivers of children under 18 with medical complexity	Adult patients
	Children with specific medical diagnoses if they meet criteria for medical complexity	Children with behavioural, emotional or mental health conditions (e.g., autism, depression, Attention Deficit Hyperactivity Disorder)
	Healthcare providers for children with medical complexity	
**Concept**	Shared decision making in the paediatric clinical context	Decisions about vaccinations or public health issues
		Pregnancy, perinatal or viability decision making
		Decision about participating in research
**Context**	Outpatients or tertiary care settings	Inpatient hospital settings
	Children's community health services	Primary care settings
	Contexts spanning multiple settings if they address paediatric community healthcare delivery	Settings outside healthcare (i.e., education)
		Universal health service
		Prevention programmes

Information sources

The search included literature published from 1982 when SDM was first mentioned in scientific literature [9]. Only articles published in English were included due to the time and cost of transcription. A three-step search strategy was followed [18]. An initial search of Medline and CINAHL identified text words and index terms to develop a full search strategy (Table 2). This search strategy was reviewed by a librarian using the Peer Review of Electronic Search Strategies (PRESS) 2015 guideline [22] and was used to search databases including Medline, CINAHL, EMBASE, AMED,

Confidential: For Review Only

PsycINFO, PubMed, Cochrane Database of Systematic Reviews and sources of grey literature including Open Grey, NICE guidelines, and CanChild website. The reference lists of included sources were screened for additional sources. The final search was completed on the 26th May 2022.

Table 2 Text words, index terms and subject heading identified for full search

Key concept	Text words / Index terms / MeSH terms (Medical Subject Headings) – combined using Boolean operators AND/OR
**Children**	child; child, preschool; adolescent; infant, extremely premature; infant; infant, newborn; paediatric
**Medical complexity**	medical complexity; special health care needs; disabilities; assistive technology; disabled children; developmental delay; chronic disease/th [therapy]; nervous system diseases/th [therapy]; medical fragility
**Shared decision-making**	parental decision-making; shared decision-making; parent perspective; decision making; patient participation; family-centred care; patient-centred care; professional-family relationship; parental discretion; bioethical issues;

Selection of evidence

Identified sources were uploaded to Covidence systematic review software and duplicates removed. Titles and abstracts were screened against criteria by (anon) and 33% was screened by (anon). Full texts of potentially relevant sources were assessed against inclusion criteria by (anon) and 33% by (anon). Reasons for exclusion of sources at full text were recorded. Disagreements between the reviewers were resolved through discussion and a third reviewer if needed (anon). The result of the search is outlined in a PRISMA flow chart (Figure 1).

Data charting process

The JBI source of evidence template [18] was modified for extraction of details about the author, publication year, country, participants, aim, context, study methods and findings relevant to the review questions. (Anon) completed data extraction and 10% were checked for consistency by (anon). Discrepancy in extraction were resolved through discussion. The extraction tool was iterative and was updated as the researchers became more familiar with the evidence.

Synthesis of results

Data was analysed by quantifying text, conducting basic qualitative content analysis and frequency counts [23]. Barriers and facilitators were ordered according to themes and mapped onto the ecological model of health behaviour (EMHB) [23,24]. The EMHB emphasizes the multiple layers of influence on healthcare behaviour and can guide the development of interventions by ensuring consideration of all factors impacting implementation [24]. Four ecological levels were used, 1) individual level including factors related to the child's needs, 2) family level relating to knowledge, attitudes, and skills of parents, 3) interpersonal level focused on interactions between HCPs and parents, and 4) organisational level considering institutional and HCP practices.

Confidential: For Review Only

Patient and public involvement statement

No patients were involved in conducting this scoping review.

RESULTS

Description of included sources

Thirty articles were included in this review (Table 3), 18 were primary studies, eleven used qualitative [4,25–34], five quantitative [3,35–38] and two mixed methods [39,40]. Eight articles were theoretical or opinion pieces [41–48] and four literature reviews [49–52]. Twelve of the primary studies included parent participants, one included HCPs and five included parents and HCPs. Seventeen sources originated in the USA, seven in Canada, four in the UK, two in the Netherlands and one in South Korea. Research interest in SDM for CMC is increasing with 23 articles published in the last ten years of which 15 were published in the last five years.

Table 3 Description of sources (ordered from earliest publication date)

Lead author	Title	Country	Date	Methods	Aim
**Bazyk, S.**	Changes in Attitudes and Beliefs Regarding Parent Participation and Home Programs: An Update	United States	1989	Text/opinion	To discuss traditional and current attitudes and practices regarding parent participation.
**Brotherson, M.**	Quality of life issues for families who make the decision to use a feeding tube for their child with disabilities	United States	1995	Qualitative	To explore the issues families face in deciding whether to place a feeding tube.
**Katz, L.**	A cultural interpretation of early intervention teams and the IFSP: parent and professional perceptions of roles and responsibilities.	United States	1995	Qualitative	To understand how members of an early intervention team involve families in developing of Individual Family Service Plans.
**Guerriere, D.N.**	Mothers' decisions about gastrostomy tube insertion in children: factors contributing to uncertainty	Canada	2003	Mixed methods	To explore mothers' perceptions of decision uncertainty.
**Blue-Banning, M.**	Dimensions of family and professional partnerships: Constructive guidelines for collaboration	United States	2004	Qualitative	To identify indicators of professional behaviour indicative of collaborative partnerships.
**Denboba, D.**	Achieving family and provider partnerships for children with special health care needs.	United States	2006	Qualitative	To assess whether families feel like partners in decision-making by their doctors.
**Arvedson, J.C.**	Ethical and legal challenges in feeding and swallowing intervention for infants and children	United States	2007	Text / opinion	To outline current state of evidence-based decision making with feeding and swallowing.
**Pickering, D.**	Disabled children's services: how do we measure family-centred care?	UK	2010	Mixed methods	To evaluate staff and parental views of family-centred care in organizations providing

Confidential: For Review Only

					services to young disabled children in Wales.
**Brotherton, A.**	Mothers' process of decision making for gastrostomy placement	UK	2012	Qualitative	To explore mothers' constructions of decision making in gastrostomy feeding.
**Kruijsen-Terpstra, A.J.A**	Parents' experiences with physical and occupational therapy for their young child with cerebral palsy: a mixed studies review.	The Netherlands	2013	Review	To review literature on the experiences of parents of children with cerebral palsy with the physical and/or occupational therapy of their child.
**Stille, C.J.**	Parent Partnerships in Communication and Decision Making About Subspecialty Referrals for Children with Special Needs	Canada	2013	Qualitative	To describe factors influencing parent-clinician partnerships in SDM when children with special health care needs are referred to subspecialists.
**An, M.**	Family-professional collaboration in pediatric rehabilitation: a practice model.	United States	2014	Text / opinion	To describe a practice model of family-professional collaboration for paediatric rehabilitation.
**Austin, D.**	Improving Partnerships to Make Family-Centered Care Work for Children with Special Health Care Needs.	United States	2014	Text / opinion	Explaining the importance of partnership working from a parental perspective
**Smalley, L.P.**	Family perceptions of shared decision-making with health care providers: results of the National Survey of Children With Special Health Care Needs, 2009-2010.	United States	2014	Quantitative	To use data from a national survey to determine families' perceptions of SDM and determine the socio-demographic correlates.
**Zaal-Schuller, I.H.**	How parents and physicians experience end-of-life decision-making for children with profound intellectual and multiple disabilities.	The Netherlands	2016	Qualitative	To compare experiences of parents and physicians involved in the end-of-life decision process.
**Adams, R.C.**	Shared Decision-Making and Children with Disabilities: Pathways to Consensus.	United States	2017	Text / opinion	To provide a basis for a systematic approach to implementation of SDM.
**Popejoy, E.**	Decision-making and future planning for children with life-limiting conditions: a qualitative systematic review and thematic synthesis.	UK	2017	Review	To synthesize findings from qualitative research about decision-making and future planning for children with life-limiting conditions.
**Lin, J.L.**	Shared Decision Making among Children with Medical Complexity: Results from a Population-Based Survey.	Canada	2018	Quantitative	To compare the rates of SDM reported by parents of CMC with the rates of SDM reported by parents of non-complex children with special health care needs.

Confidential: For Review Only

**Madrigal, V.N.**	Supporting Family Decision-making for a Child Who Is Seriously Ill: Creating Synchrony and Connection	United States	2018	Text / opinion	To discuss the process of supporting families facing chronic and serious illness during decision-making.
**Mahant, S.**	Decision-making around gastrostomy tube feeding in children with neurologic impairment: Engaging effectively with families	Canada	2018	Text / opinion	To review evidence and conceptual frameworks and provide recommendations to support decisions about gastrostomies.
**Jolles, M.P.**	Shared decision-making and parental experiences with health services to meet their child's special health care needs: Racial and ethnic disparities.	United States	2018	Quantitative	To test the relationship between SDM and parental report of frustration with efforts to get services for their child and to assess SDM's influence on minority parents' service experiences.
**An, M.**	Effects of a Collaborative Intervention Process on Parent-Therapist Interaction: A Randomized Controlled Trial.	South Korea	2019	Quantitative	To determine whether collaborative intervention impacted interactions between parents of children with physical disabilities and physical therapists.
**Buchanan, F.**	What makes difficult decisions so difficult?: An activity theory analysis of decision making for physicians treating children with medical complexity.	Canada	2020	Qualitative	To first understand the complexity of the activity of decision-making.
**Lee, J.K.**	Decision-Making for Children with Medical Complexity: The Role of the Primary Care Pediatrician.	United States	2020	Text / opinion	Discussion of influences on decision-making from a paediatrician's perspective.
**Lin, J.L.**	Parent Perspectives in Shared Decision-Making for Children with Medical Complexity.	United States	2020	Qualitative	To identify components of SDM unique to the care of CMC from the perspective of parents.
**Jabre, N.A.**	Parent perspectives on facilitating decision-making around pediatric home ventilation.	United States	2021	Qualitative	To understand parent perspectives about how clinicians can better facilitate decision-making around home ventilation.
**Reeder, J.**	Becoming an empowered parent. How do parents successfully take up their role as a collaborative partner in their child's specialist care?	UK	2021	Qualitative	To explore the important themes of dis/empowerment and the influence of the therapeutic relationship.
**Buchanan, F**	Decision-making for Parents of Children With Medical Complexities: Activity Theory Analysis.	Canada	2022	Qualitative	To explore decision-making of parents of CMC as an activity within the context of a process shared between clinician and parent.

Confidential: For Review Only

**Jonas, D.**	Parental Decision-Making for Children With Medical Complexity: An Integrated Literature Review.	United States	2022	Review	To consolidate existing literature on parental experience of medical decision making for CMC.
**LeGrow, K.R.N.**	Relational Aspects of Parent and Home Health Care Provider Care Practices for Children With Complex Care Needs Receiving Health Care Services in the Home: A Narrative Review.	Canada	2022	Review	To review literature on relational aspects of parent and home healthcare provider care practices for children with complex healthcare needs.

Definition of shared decision-making for children with medical complexity

Eighteen articles defined or described SDM (Figure 2). A collaborative approach and equal partnership between parents and HCPs were most frequently noted as key elements of SDM. Most other elements offered guidance on how to achieve this partnership. Three sources referenced the impact of uncertainty of prognosis and treatment outcomes for CMC on this collaborative process.

Implementation of shared decision-making for children with medical complexity in community health services

Two qualitative [26,34] and two quantitative [3,36] studies reported on the implementation of SDM with CMC in community settings. The two qualitative studies were conducted 26 years apart (ethnographic study in 1995 [26] and interview-based study in 2021 [34]), in both parents perceived a power imbalance between them and HCPs with decision-making situated with HCPs. Two quantitative studies analysed the same dataset from a national survey in the USA [3,36] and found that although 85% of parents felt like partners with their child's doctor, there was a negative association with minority ethnic and low socio-economic status [36] and children with greater complexity [3]. This was attributed to multidisciplinary support needs, frequent hospital admissions, clinical uncertainty and social difficulties often experienced by parents of CMC.

Differences in shared decision-making in different communities

Eight of the eighteen primary studies reported on participant ethnicity but only three USA studies reported on differences in SDM among participants from a Black, Hispanic, and White background [35,36,38]. These studies found that families from minority ethnic backgrounds, those with lower educational backgrounds and lower income levels experienced less coordinated care and less SDM.

Barriers and facilitators to shared decision-making for children with medical complexity

Twenty-eight articles mentioned at least one barrier or facilitator to SDM. The most cited barriers related to clinical uncertainty of CMC, power imbalance between parents and HCPs and the lack of continuity of care (Table 4).

Table 4 Barriers to SDM for CMC mapped to the ecological model

**INDIVIDUAL (CHILD) LEVEL – theme: uncertainty**

Confidential: For Review Only

Uncertainty about diagnosis or clinical management options	[3,28,29,31,32,36,45,47,48,50]
Lack of evidence and uncertain illness trajectories	[3,25,31,32,45,47,50]
Limited or conflicting information	[25,29,30,39]
Uncertainty about child's comfort and quality if life	[36,50]
**FAMILY LEVEL**	
Language barriers	[29,36,38,40,45]
Poor general and health literacy	[25,28,45,50]
Lack of parental understanding of child's diagnosis and prognosis	[29,40,45]
Lack of trust in HCPs	[27,34,50]
Parents not feeling heard	[34,44,50]
Poverty, Black or minority ethnic background	[35,36]
Parental physical and emotional exhaustion and strong emotions	[32,50]
**INTERPERSONAL LEVEL – theme: power imbalance**	
Hierarchy and power imbalance, coercive conversations by HCP, failure to explain options fully or withholding information and labelling parents as non-compliant if they disagree with HCPs	[3,4,26,28–30,32,34,36,44,45,48,50]
Using medical jargon and providing too detailed information	[4,27,33,40,43,45,51]
HCPs not valuing parental opinion and experience	[30,32,34,45]
**ORGANISATIONAL LEVEL – theme: lack of continuity of care**	
Involvement of multiple subspecialities, lack of continuity of care	[3,4,32,40,44,45,48,50]
Healthcare systems that dictate treatment options	[4,31,32,50]
Lack of time in consultations	[3,43,45]
Lack of professional understanding and valuing of SDM	[31,45]
Limited access to the right person to answer questions	[4,50]

The most cited facilitators to SDM for CMC included sharing of accessible, adequate, accurate and balanced information about all treatment options including knowing about uncertainty. Several facilitators related to the way HCPs viewed and engaged parents as active team members, service accessibility and attributes relating to the family (Table 5).

Table 5 Facilitators to SDM for CMC

**INDIVIDUAL (CHILD) LEVEL**	
Valuing the personhood of the child	[27,33,51]
**FAMILY LEVEL**	
Parental comfort with decision-making	[28,32,38,50,52]
Knowledge of the healthcare system	[4,50,51]
Parental educational level	[31,50]
Parental understanding of the child's diagnosis and prognosis	[34,50]
**INTERPERSONAL LEVEL**	
Mutual trust and respect	[4,27,29,35,46,48,49]
Actively empowering families to express their opinions, fears and hopes	[27,28,40,46,47,49]
Reciprocal good Interpersonal skills	[27,30,31,40,50]
Reciprocal active listening	[27,34,35,40,46]
Regarding parents as experts on their child	[29,40,49–51]
Having shared goals	[27,44,46,48]
Sensitivity to cultural differences	[27,33,40,50]
Showing dedication to the family	[27,33,51]
Professional awareness of parental decision-making preferences	[46,47]
Respecting family decisions	[26,31]

Confidential: For Review Only

ORGANISATIONAL LEVEL – theme: information and access	
Having accessible, sufficient, accurate and balanced information about all treatment options including knowing about uncertainty	[4,25–27,30,32,33,46,47,51]
Access to peer-to-peer support	[25,29,33,36,38,47,50]
Having sufficient time to consider information and knowing the time-horizon for decision-making	[30,32,33,47,50,51]
Access to information from non-professional sources such as social media	[31,32,39,47,50]
Including parents as members of the team	[27,36,40]
Continuity of care	[33,40,51]
Access to interpreters if needed	[29,40]
Access to the right healthcare professionals to answer questions	[4,29]
HCP seeking advice from the wider team in the face of uncertainty	[31,51]
Having access to written information	[29,30]

Research recommendations

Nineteen articles concluded with research recommendations, most related to discovering how to involve families and develop collaborative relationships, particularly families from diverse backgrounds (Table 6).

Table 6 Research recommendations

Discover how to involve families and develop collaborative relationships in SDM	[26,27,30,31,34,49,50,52]
Explore SDM within diverse communities, including diversity in family structure, culture and ethnicity, education level and healthcare setting	[30,32,36–38,49,51,52]
Explore family and healthcare professional's beliefs, perspectives, and experiences of SDM	[30–32,43,51,52]
Develop guidelines for SDM	[27,36,46,50]
Evaluate the effect of professional training on SDM	[27,34,37]
Evaluate proposed models of SDM	[36,43]
Develop outcomes measures for SDM	[45]
Develop support technologies for SDM	[45]
Investigate information needs for effective SDM	[25]

DISCUSSION

This scoping review explored the landscape of SDM for CMC in community health services. SDM is important in this population due to the complex long-term nature of their health conditions and high healthcare use. Like previous reviews [6,10], this review found no unifying definition for SDM in the literature. All sources highlighted the importance of SDM; however, few studies explored the effectiveness of SDM for CMC. The lack of implementation research is a shortcoming in paediatric research generally [10] with evidence mainly pertaining to adult care [5,17]. Limited research exists about SDM for CMC who are from a minority ethnic or disadvantaged background. Three studies showed poorer implementation in Black and Hispanic communities in the USA [35,36,38]. This is congruent with evidence showing that adults from minority ethnic or disadvantaged backgrounds experience more barriers to SDM [53]. Research shows that SDM interventions can significantly improve the outcomes for disadvantaged adult patients, including increased knowledge and participation in decision-making [54]. It is therefore not surprising that exploring SDM in diverse communities was one of the most cited research recommendations.

Confidential: For Review Only

Many influencing factors could either be barriers or facilitators to SDM depending on the context [5,7,53]. Most factors identified in this review are situated in the organisational level where HCPs can influence change. This include organisational and personal understanding of and commitment to SDM, interpersonal skills that build trust and respect, active listening, cultural sensitivity, empowerment of families to be active team members and to share their values, opinions and fears, continuity of care, access to the right HCPs and enough time in consultations. Previous studies found that the top patient-reported barriers to SDM include disorganised healthcare systems and the quality of interactions with HCPs [53], whereas the main barriers identified by HCPs are lack of time and motivation to pursue SDM and a perception that patients do not want to engage in decision-making [5,7]. There is however an awareness amongst HCPs that good communication and coordination of care can improve SDM [2]. To improve implementation of SDM it should be viewed as a culture within organisations and a way of interacting with every service-user rather than another clinical tool [2,10].

Another significant patient-identified barrier is hierarchy and power-imbalance still prevalent in traditional approaches, where HCPs are the main decision-makers [8]. Even when espousing SDM, HCPs often provide detailed information about their preferred treatment option first to convince or coerce the patient [7]. This review found that HCPs often fail to explain all available options, withhold information, and use jargon. In some instances, HCPs provide too much and too detailed information, also negatively impacting the decision-making process. Effective information exchange is an important step in the decision-making process [55] and can either decrease or increase the power-balance in the relationship.

This power-imbalance is compounded by clinical information being held by HCPs who can influence the accuracy, clarity, tailoring and sharing of that information [7,13,53]. This review identified accessible, adequate, accurate and balanced information as one of the most significant contributors to successful SDM and conversely, the lack of evidence and information as a barrier to SDM. Providing information that outlines options, risks and uncertainties can improve SDM [7,8] and if presented in an accessible and culturally sensitive format, can help overcome language and socio-economic barriers such as poor literacy [53].

A complicating factor in SDM for CMC is however the lack of clinical and empirical evidence and information due to the unique illness trajectory of CMC, leading to high levels of uncertainty. The presence of uncertainty was the most striking barrier to SDM for CMC found in this review and sets CMC apart from many other patient populations. A high level of uncertainty is not a typical feature of SDM for children [7] or adults [53] but has been cited in areas such as neonatal intensive care [15], paediatric end-of-life care [15,56] and dementia end-of-life care [55]. The similarities with these clinical areas underscore the high-stress nature of decision-making for CMC [13,14].

This review highlights the need for further research to increase the evidence-base relating to diagnosis, prognosis, and treatment options for CMC and to address the implementation of SDM for CMC, specifically focussing on families from diverse backgrounds who often experience less SDM.

Strengths and limitations

This review contributes to the limited evidence-base concerning SDM for CMC and highlights themes around uncertainty, power-imbalance, and information sharing on implementation of SDM. The risk of missing sources due to the number of synonymous terms for SDM [6] and CMC [1] used in the literature was mitigated by developing a comprehensive list of search terms and conducting a systematic search using a range of databases. Results were strengthened by having second reviewers at all screening stages. Most sources originated in the USA and Canada where barriers

Confidential: For Review Only

might be different to the UK and other parts of the world. The studies that included minority ethnic groups were conducted in the USA and do not represent the UK population.

CONCLUSION

This scoping review revealed that uncertainty about diagnosis, prognosis, and treatment outcomes for CMC has a significant impact on SDM, in addition to barriers and facilitators identified in other paediatric and adult populations, highlighting the need to advance the clinical evidence base for this population. Furthermore, many factors impacting SDM fall within the organisational level where HCPs can influence change, including pursuing a power-balance and equal partnership, improving continuity of care, and improving information resources to meet the needs of parents of CMC, including those from diverse backgrounds. Focusing on these factors can potentially improve medical and developmental outcomes, quality of life of children and families and more effective use of healthcare resources. This review can be used to guide a research strategy in the field of SDM for CMC in community health services.

Contributors: SJ, ND and CS planned the study. SJ is the guarantor and took the lead in performing the scoping literature search and data analysis and writing the manuscript. KB, JO, and KS were second reviewers for source selection and extraction and reviewed the manuscript. ND and CS supervised the study and reviewed the manuscript. All authors critically reviewed the final draft of the manuscript.

Funding: SJ is an ICA Pre-doctoral Clinical Academic Fellow supported by Health Education England and the National Institute for Health Research, grant number: NIHR301944. The views expressed in this publication are those of the authors and not necessarily those of the NHS, the National Institute for Health Research or the Department of Health and Social Care.

Competing interests: None

Patient consent for publication: Not applicable

Data availability statement: Not applicable

References

1 Cohen E, Kuo DZ, Agrawal R, *et al.* Children with medical complexity: An emerging population for clinical and research initiatives. Pediatrics. 2011;**127**:529–38. doi:10.1542/peds.2010-0910

2 Birchley G, Thomas-Unsworth S, Mellor C, *et al.* Factors affecting decision-making in children with complex care needs: a consensus approach to develop best practice in a UK children's hospital. *BMJ Paediatr Open* 2022;**6**. doi:10.1136/bmjpo-2022-001589

Confidential: For Review Only

3 Lin JL, Cohen E, Sanders LM. Shared Decision Making among Children with Medical Complexity: Results from a Population-Based Survey. *Journal of Pediatrics* 2018;**192**:216–22. doi:http://dx.doi.org/10.1016/j.jpeds.2017.09.001

4 Buchanan F, Lai C, Cohen E, *et al.* Decision-making for Parents of Children With Medical Complexities: Activity Theory Analysis. *J Particip Med* 2022;**14**:e31699. doi:https://dx.doi.org/10.2196/31699

5 Gravel K, Légaré F, Graham ID. Barriers and facilitators to implementing shared decision-making in clinical practice: A systematic review of health professionals' perceptions. Implementation Science. 2006;**1**. doi:10.1186/1748-5908-1-16

6 Park ES, Cho IY. Shared decision-making in the paediatric field: a literature review and concept analysis. Scand J Caring Sci. 2018;**32**:478–89. doi:10.1111/scs.12496

7 Boland L, Graham ID, Légaré F, *et al.* Barriers and facilitators of pediatric shared decision-making: A systematic review. Implementation Science. 2019;**14**. doi:10.1186/s13012-018-0851-5

8 Coulter Angela, Collins A, King's Fund Centre (London). *Making shared decision-making a reality: no decision about me, without me*. King's Fund 2011. https://www.kingsfund.org.uk/publications/making-shared-decision-making-reality (accessed 16 Oct 2022).

9 Mulley AG, Trimble Chris, Elwyn Glyn. *Patients' preferences matter: stop the silent misdiagnosis*. King's Fund 2012. https://www.kingsfund.org.uk/publications/patients-preferences-matter (accessed 10 Aug 2022).

10 Wyatt KD, List B, Brinkman WB, *et al.* Shared Decision Making in Pediatrics: A Systematic Review and Meta-analysis. *Acad Pediatr* 2015;**15**:573–83. doi:10.1016/j.acap.2015.03.011

11 Vemuri S, Hynson J, Williams K, *et al.* Decision-making approaches for children with life-limiting conditions: results from a qualitative phenomenological study. *BMC Med Ethics* 2022;**23**. doi:10.1186/s12910-022-00788-7

12 Fiks AG, Hughes CC, Gafen A, *et al.* Contrasting parents' and pediatricians' perspectives on shared decision-making in ADHD. *Pediatrics* 2011;**127**. doi:10.1542/peds.2010-1510

13 Dias BC, Marcon SS, Reis P dos, *et al.* Family dynamics and social network of families of children with special needs for complex/continuous cares. *Rev Gaucha Enferm* 2020;**41**:e20190178. doi:10.1590/1983-1447.2020.20190178

14 Woodgate RL, Edwards M, Ripat JD, *et al.* Intense parenting: A qualitative study detailing the experiences of parenting children with complex care needs. *BMC Pediatr* 2015;**15**. doi:10.1186/s12887-015-0514-5

15 Krick JA, Hogue JS, Reese TR, *et al.* Uncertainty: An Uncomfortable companion to decision-making for infants. *Pediatrics* 2020;**146**:S14–7. doi:10.1542/peds.2020-0818E

16 Jacobs S, Davies N, Smith CH. Shared decision-making for children with medical complexity in community health services – scoping review protocol. *Open Science Framework* Published Online First: 2022. doi:DOI 10.17605/OSF.IO/VJ2KB

17 Joseph-Williams N, Lloyd A, Edwards A, *et al.* Implementing shared decision making in the NHS: lessons from the MAGIC programme. *BMJ* 2017;**357**:j1744. doi:10.1136/bmj.j1744

Confidential: For Review Only

18 Peters MDJ, Marnie C, Tricco AC, *et al.* Updated methodological guidance for the conduct of scoping reviews. *JBI Evid Synth* 2020;**18**:2119–26. doi:10.11124/JBIES-20-00167

19 Peters MDJ, Godfrey C, Mcinerney P, *et al.* Best practice guidance and reporting items for the development of scoping review protocols. *JBI Evid Synth* 2022;**20**:1–19. doi:10.11124/JBIES-21-00242

20 Jordan PZ. JBI Manual for Evidence Synthesis. 2020. doi:10.46658/jbimes-20-01

21 Tricco AC, Lillie E, Zarin W, *et al.* PRISMA extension for scoping reviews (PRISMA-ScR): Checklist and explanation. Ann Intern Med. 2018;**169**:467–73. doi:10.7326/M18-0850

22 McGowan J, Sampson M, Salzwedel DM, *et al.* PRESS Peer Review of Electronic Search Strategies: 2015 Guideline Statement. *J Clin Epidemiol* 2016;**75**:40–6. doi:10.1016/j.jclinepi.2016.01.021

23 Pollock D, Peters MDJ, Khalil H, *et al.* Recommendations for the extraction, analysis, and presentation of results in scoping reviews. JBI Evid Synth. [preprint] October 14, 2022 doi: 10.11124/JBIES-22-00123. *Ashrita Saran* 2022;**10**:18. doi:10.11124/JBIES-22-00123

24 Sallis JF, Owen N, Fisher E. Ecological Models of Health Behavior. In: Glanz K, Rimer BK, Viswanath K, eds. *Health Behavior and Health Education: Theory, Research and Practice*. Jossey-Bass 2008. 465–86.https://www.researchgate.net/publication/43508113 (accessed 28 Nov 2022).

25 Brotherson MJ, Oakland MJ, Secrist-Mertz C, *et al.* Quality of Life Issues for Families Who Make the Decision to Use a Feeding Tube for Their Child With Disabilities. *Journal of the Association for Persons with Severe Handicaps* 1995;**20**:202–12. doi:doi.org/10.1177/154079699502000305

26 Katz L, Scarpati S. A Cultural Interpretation of Early Intervention Teams and the IFSP: Parent and Professional Perceptions of Roles and Responsibilities. *Infant-Toddler Intervention: The Transdisciplinary Journal* 1995;**5**:177–91.https://www.researchgate.net/publication/234649683

27 Blue-Banning M, Summers JA, Frankland HC, *et al.* Dimensions of family and professional partnerships: Constructive guidelines for collaboration. *Except Child* 2004;**70**:167–84. doi:10.1177/001440290407000203

28 Brotherton A, Abbott J. Mothers' process of decision making for gastrostomy placement. *Qual Health Res* 2012;**22**:587–94. doi:10.1177/1049732311423841

29 Stille CJ, Fischer SH, Nancy ;, *et al.* Parent Partnerships in Communication and Decision Making About Subspecialty Referrals for Children With Special Needs. *Acad Pediatr* 2013;**13**:122–32. doi:DOI: 10.1016/j.acap.2012.12.003

30 Zaal-Schuller, Willems DL, Ewals FVPM, *et al.* How parents and physicians experience end-of-life decision-making for children with profound intellectual and multiple disabilities. *Res Dev Disabil* 2016;**59**:283–93. doi:https://dx.doi.org/10.1016/j.ridd.2016.09.012

31 Buchanan F, Cohen E, Milo-Manson G, *et al.* What makes difficult decisions so difficult?: An activity theory analysis of decision making for physicians treating children with medical complexity. *Patient Educ Couns* 2020;**103**:2260–8. doi:10.1016/j.pec.2020.04.027

Confidential: For Review Only

32 Lin, Clark CL, Halpern-Felsher B, *et al.* Parent Perspectives in Shared Decision-Making for Children With Medical Complexity. *Acad Pediatr* 2020;**20**:1101–8. doi:10.1016/j.acap.2020.06.008

33 Jabre NA, Raisanen JC, Shipman KJ, *et al.* Parent perspectives on facilitating decision-making around pediatric home ventilation. *Pediatr Pulmonol* 2022;**57**:567–75. doi:10.1002/ppul.25749

34 Reeder J, Morris J. Becoming an empowered parent. How do parents successfully take up their role as a collaborative partner in their child's specialist care? *Journal of Child Health Care* 2021;**25**:110–25. doi:10.1177/1367493520910832

35 Denboba D, McPherson MG, Kenney MK, *et al.* Achieving family and provider partnerships for children with special health care needs. *Pediatrics* 2006;**118**:1607–15. doi:DOI: 10.1542/peds.2006-0383

36 Smalley, Kenney M, Denboba D, *et al.* Family Perceptions of Shared Decision-Making with Health Care Providers: Results of the National Survey of Children with Special Health Care Needs, 2009-2010. *Matern Child Health J* 2014;**18**:1316–27. doi:10.1007/s10995-013-1365-z

37 An M, Palisano RJ, Yi CH, *et al.* Effects of a Collaborative Intervention Process on Parent-Therapist Interaction: a Randomized Controlled Trial. *Phys Occup Ther Pediatr* 2019;**39**:259-275. doi:10.1080/01942638.2018.1496965

38 Jolles MP, Lee PJ, Javier JR. Shared decision-making and parental experiences with health services to meet their child's special health care needs: Racial and ethnic disparities. *Patient Educ Couns* 2018;**101**:1753–60. doi:10.1016/j.pec.2018.05.022

39 Guerriere DN, Mckeever P, Llewellyn-Thomas H, *et al.* Mothers' decisions about gastrostomy tube insertion in children: factors contributing to uncertainty. *Dev Med Child Neurol* 2003;**45**:470–6. doi:10.1017/S0012162203000872

40 Pickering D, Busse M. Disabled children's services: How do we measure family-centred care? *Journal of Child Health Care* 2010;**14**:200–7. doi:10.1177/1367493509360207

41 Arvedson JC, Lefton-Greif MA. Ethical and legal challenges in feeding and swallowing intervention for infants and children. *Semin Speech Lang* 2007;**28**:232–8. doi:10.1055/s-2007-984729

42 Bazyk S. Changes in Attitudes and Beliefs Regarding Parent Participation and Home Programs: An Update. *American Journal of Occupational Therapy* 1989;**43**:723–8. doi:10.5014/ajot.43.11.723

43 An M, Palisano RJ. Family-professional collaboration in pediatric rehabilitation: a practice model. *Disabil Rehabil* 2014;**36**:434–40. doi:10.3109/09638288.2013.797510

44 Austin D. Improving Partnerships to Make Family-Centered Care Work for Children with Special Health Care Needs. Science of Caring. 2014;:1.https://scienceofcaring.ucsf.edu/community/improving-partnerships-make-family-centered-care-work-children-special-health-care-needs (accessed 2 Mar 2022).

45 Adams, Levy SE. Shared Decision-Making and Children With Disabilities: Pathways to Consensus. *Pediatrics* 2017;**139**:1–9.https://doi.org/10.1542/peds.2017-0956

Confidential: For Review Only

46 Madrigal VN, Patterson Kelly K, Washington G. Supporting Family Decision-making for a Child Who Is Seriously Ill: Creating Synchrony and Connection. *Pediatrics* 2018;**142**:S170–7. doi:10.1542/peds.2018-0516H

47 Mahant S, Cohen E, Nelson KE, *et al.* Decision-making around gastrostomy tube feeding in children with neurologic impairment: Engaging effectively with families. *Paediatrics and Child Health (Canada)* 2018;**23**:209–13. doi:10.1093/pch/pxx193

48 Lee KJ, Hill DL, Feudtner C. Decision-making for children with medical complexity: The role of the primary care pediatrician. *Pediatr Ann* 2020;**49**:e473–7. doi:10.3928/19382359-20201013-01

49 Kruijsen-Terpstra AJ, Ketelaar M, Boeije H, *et al.* Parents' experiences with physical and occupational therapy for their young child with cerebral palsy: a mixed studies review. *Child Care Health Dev* 2014;**40**:787–96. doi:http://dx.doi.org/10.1111/cch.12097

50 Popejoy E, Pollock K, Almack K, *et al.* Decision-making and future planning for children with life-limiting conditions: a qualitative systematic review and thematic synthesis. *Child Care Health Dev* 2017;**43**:627–44. doi:10.1111/cch.12461

51 Jonas, Scanlon C, Bogetz JF. Parental Decision-Making for Children With Medical Complexity: An Integrated Literature Review. *J Pain Symptom Manage* 2022;**63**. doi:10.1016/j.jpainsymman.2021.07.029

52 LeGrow, Cohen E, Espin S. Relational Aspects of Parent and Home Health Care Provider Care Practices for Children With Complex Care Needs Receiving Health Care Services in the Home: A Narrative Review. *Acad Pediatr* 2022;**22**:196–202. doi:10.1016/j.acap.2021.08.009

53 Joseph-Williams N, Elwyn G, Edwards A. Knowledge is not power for patients: A systematic review and thematic synthesis of patient-reported barriers and facilitators to shared decision making. Patient Educ Couns. 2014;**94**:291–309. doi:10.1016/j.pec.2013.10.031

54 Durand MA, Carpenter L, Dolan H, *et al.* Do interventions designed to support shared decision- making reduce health inequalities? A systematic review and meta-analysis. *PLoS One* 2014;**9**. doi:10.1371/journal.pone.0094670

55 Davies N, de Souza T, Rait G, *et al.* Developing an applied model for making decisions towards the end of life about care for someone with dementia. *PLoS One* 2021;**16**. doi:10.1371/journal.pone.0252464

56 Richards CA, Starks H, O'Connor MR, *et al.* Physicians Perceptions of Shared Decision-Making in Neonatal and Pediatric Critical Care. *American Journal of Hospice and Palliative Medicine* 2018;**35**:669–76. doi:10.1177/1049909117734843

Figures:

Figure 1 PRISMA flow chart

Figure 2 Elements of shared decision-making for children with medical complexity

Confidential: For Review Only

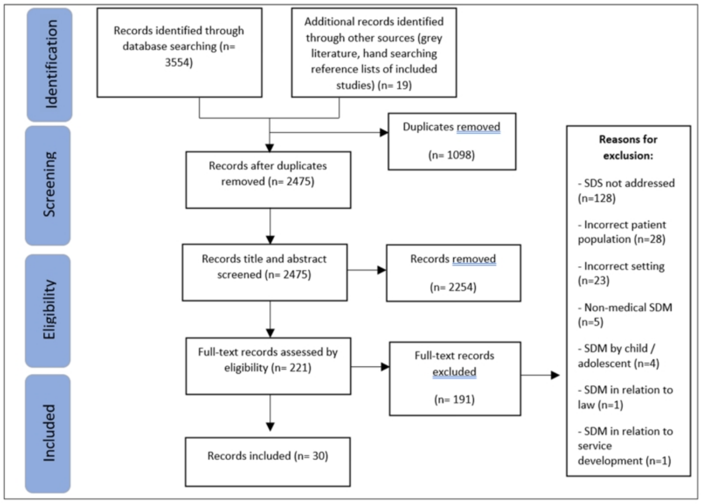

Figure 1 PRISMA flow chart

533x754mm (118 x 118 DPI)

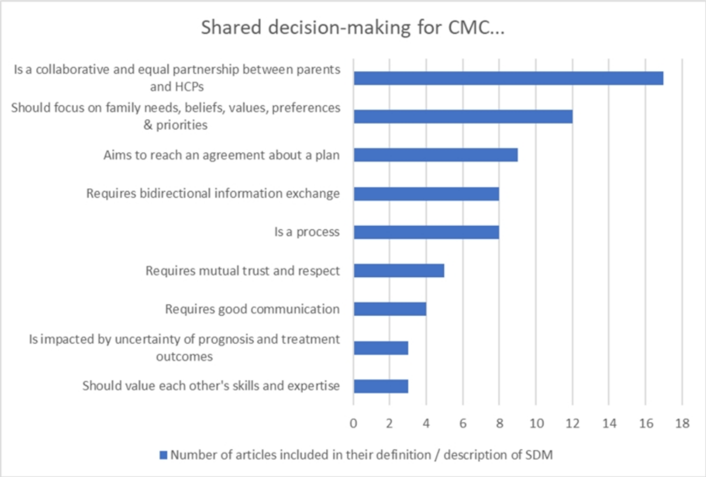

Figure 2 Elements of shared decision-making for children with medical complexity

533x754mm (118 x 118 DPI)

BMJ Paediatrics Open

Shared decision-making for children with medical complexity in community health services – a scoping review

Journal:	*BMJ Paediatrics Open*
Manuscript ID	bmjpo-2023-001866.R1
Article Type:	Original research
Date Submitted by the Author:	27-Feb-2023
Complete List of Authors:	Jacobs, Sonja; Barts Health NHS Trust<br>Davies, Nathan; University College London, Research Department of Primary Care & Population Health<br>Butterick, Katherine L; Barts Health NHS Trust<br>Oswell, Jane L; Barts Health NHS Trust<br>Siapka, Konstantina; Barts Health NHS Trust<br>Smith, Christina; University College London
Keywords:	Health services research

SCHOLARONE™
Manuscripts

BMJ

I, the Submitting Author has the right to grant and does grant on behalf of all authors of the Work (as defined in the below author licence), an exclusive licence and/or a non-exclusive licence for contributions from authors who are: i) UK Crown employees; ii) where BMJ has agreed a CC-BY licence shall apply, and/or iii) in accordance with the terms applicable for US Federal Government officers or employees acting as part of their official duties; on a worldwide, perpetual, irrevocable, royalty-free basis to BMJ Publishing Group Ltd ("BMJ") its licensees and where the relevant Journal is co-owned by BMJ to the co-owners of the Journal, to publish the Work in this journal and any other BMJ products and to exploit all rights, as set out in our licence.

The Submitting Author accepts and understands that any supply made under these terms is made by BMJ to the Submitting Author unless you are acting as an employee on behalf of your employer or a postgraduate student of an affiliated institution which is paying any applicable article publishing charge ("APC") for Open Access articles. Where the Submitting Author wishes to make the Work available on an Open Access basis (and intends to pay the relevant APC), the terms of reuse of such Open Access shall be governed by a Creative Commons licence – details of these licences and which Creative Commons licence will apply to this Work are set out in our licence referred to above.

Other than as permitted in any relevant BMJ Author's Self Archiving Policies, I confirm this Work has not been accepted for publication elsewhere, is not being considered for publication elsewhere and does not duplicate material already published. I confirm all authors consent to publication of this Work and authorise the granting of this licence.

Confidential: For Review Only

Shared decision-making for children with medical complexity in community health services – a scoping review

Sonja Jacobs[1], Nathan Davies[2], Katherine L Butterick[1], Jane L Oswell[1], Konstantina Siapka[1], Christina H Smith[3]

1, Barts Health NHS Trust, London, UK
2. Research Department of Primary Care & Population Health, University College London, UK
3 Division of Psychology and Language Sciences, University College London, UK

Corresponding author: Christina H Smith, Division of Psychology and Language Sciences, University College London, UK; christina.smith@ucl.ac.uk

Word count: 2499

Keywords:
MeSH headings:

- Decision making, shared
- Professional-Family Relations
- Disabled Children
- Community Health Services

Confidential: For Review Only

ABSTRACT

Background: Children with medical complexity is an increasing population whose parents and healthcare providers face multiple decisions. Shared decision-making is a process where patients, their families and healthcare providers collaborate to make decisions based on clinical evidence and informed preferences of the family. Shared decision-making has benefits for the child, family and healthcare providers, including improved parental understanding of the child's difficulties, increased participation, improved coping skills and more efficient healthcare use. It is however poorly implemented.

Aims and methods: A scoping review was conducted to explore shared decision-making for children with medical complexity in community health services, including how shared decision-making is defined in research, how it is implemented, including barriers and facilitators and recommendations for research. Six databases were systematically searched for papers published in English up to May 2022: Medline, CINAHL, EMBASE, PsycINFO, PubMed, Cochrane Database of Systematic Reviews, and sources of grey literature. The review is reported according to the Preferred Reporting Items for Scoping Reviews.

Results: Thirty sources met the inclusion criteria. Most factors can either be a facilitator or barrier to shared decision-making depending on the context. Two significant barriers to shared decision-making in this population include uncertainty about the child's diagnosis, prognosis, and treatment options and the presence of hierarchy and power-imbalance during clinical encounters with healthcare providers. Further influencing factors include continuity of care, the availability of accurate, accessible, adequate, and balanced information and the interpersonal and communication skills of parents and healthcare providers.

Conclusion: Uncertainty about diagnosis, prognosis, and treatment outcomes for children with medical complexity are additional challenges to the known barriers and facilitators to shared decision-making in community health services. Effective implementation of shared decision-making requires advancement of the evidence-base for children with medical complexity, reducing power-imbalance in clinical encounters, improving continuity of care, and improving the availability and accessibility of information resources.

What is already known on this topic

Shared decision-making is an evidence-based approach with known benefits to children with medical complexity, their families, and the healthcare system, it is however poorly implemented.

What this study adds

This study highlights the impact of uncertainty of diagnosis, prognosis, and treatment outcomes for children with medical complexity on shared decision-making. It highlights how healthcare providers can improve the implementation of shared decision-making by addressing the power-imbalance in clinical encounters, improving continuity of care, improving communication and interpersonal skills, and making information more accessible to parents from diverse backgrounds.

How this study might affect research, practice, or policy

This review can guide a research strategy in the field of shared decision-making for children with medical complexity in community health services support healthcare professionals to consider their influence on the decision-making process in everyday practice.

Confidential: For Review Only

INTRODUCTION

Children with medical complexity (CMC) have needs in four domains: 1) substantial family-identified healthcare and special educational needs, 2) one or more severe and potentially lifelong chronic conditions, 3) limitations to body structure and function, performance of activities and participation that may require technological assistance such as feeding tubes, and 4) high projected healthcare use including the involvement of multiple subspecialties [1]. These children are increasing in number [1,2]. Parents and healthcare providers (HCPs) for CMC face multiple, complex decisions throughout their childhood including decisions about tube feeding, mechanical ventilation, medications, and surgery [1,3,4].

Shared decision-making (SDM) is an evidence-based approach that is an essential part of patient-centred care [5–7]. It is a process where parents, as surrogate decision-makers for their child, and HCPs work in partnership to make decisions based on clinical evidence and family preferences [7–10]. This approach is supported by policy makers and regulatory bodies nationally and internationally [5–10]. The benefits for patients, families, and HCPs include improved patient or carer knowledge and understanding, reduced decisional conflict, increased participation and engagement in care, improved coping skills, and efficient use of healthcare resources [5,6,8,10]. SDM is however poorly defined due to the interpretive nature of what is meant by "shared" [6,11], with fundamental differences in how patients, carers and HCPs understand the purpose of and their role in SDM [6,8,12–14].

The difference between parental and HCP approaches to decision making often result in poor implementation of SDM. HCPs base their decisions on clinical and empirical evidence [8] which is often lacking for CMC [1,15]. Parents consider the social, emotional, and psychological impact of decisions on their child, their family and cultural and religious beliefs in addition to potential clinical outcomes [8,11,13]. Parental decisions about what is 'good enough' for their child with medical complexity are often more intensely scrutinised by HCPs than for non-medically complex children, with a lack of awareness or importance given to the impact of decisions on the family [8,11,13–16]. Clinical uncertainty combined with complex family dynamics require HCPs to swap traditional hierarchical and paternalistic approaches to decision-making, where decisions are made based on clinical information and empirical evidence [5,8,11,12,15,17], for an approach that allows parental collaboration and discretion in decision-making [11,15].

The personal and healthcare cost of poor implementation of SDM is amplified in the CMC population due to their significant healthcare use. Understanding factors impacting SDM for CMC will help to improve medical and developmental outcomes, quality of life of children and families and effective use of healthcare resources [5,8,10].

This scoping review aimed to explore the landscape of shared decision-making for children with medical complexity in community health services.

The objectives for this review were to:

1) Explore how SDM is defined in research.

2) Understand to what extent SDM is implemented for CMC in community health services.

3) Consider the differences in SDM between ethnic groups.

4) Identify the barriers and facilitators to SDM for CMC.

5) Provide recommendations for future research.

Confidential: For Review Only

METHODS

A scoping review was conducted following the Joanna Brigs Institute (JBI) manual for evidence synthesis [18–20] and was reported according to the Preferred Reporting Items for Systematic Reviews and Meta-Analyses extension for scoping reviews (PRISMA-ScR) [21].

Protocol and registration

A scoping review protocol was registered on Open Science Framework on 19-05-2022 [16].

Eligibility criteria

Eligibility criteria are outlined in Table 1. SDM included any process involving parents or caregivers in medical decision-making with HCPs [10] and included family-centred practices. Sources reporting on multiple populations or settings were included if results were reported separately or if at least 50% of the results related to the eligible population or setting. Primary research using any methodology, secondary research including systematic reviews, literature, and scoping reviews and editorial or opinion pieces were included.

Table 1 Eligibility criteria

	Included	Excluded
**Population**	Parents and/or caregivers of children under 18 with medical complexity	Adult patients
	Children with specific medical diagnoses if they meet criteria for medical complexity	Children with behavioural, emotional or mental health conditions (e.g., autism, depression, Attention Deficit Hyperactivity Disorder)
	Healthcare providers for children with medical complexity	
**Concept**	Shared decision making in the paediatric clinical context	Decisions about vaccinations or public health issues
		Pregnancy, perinatal or viability decision making
		Decision about participating in research
**Context**	Outpatients or tertiary care settings	Inpatient hospital settings
	Children's community health services	Primary care settings
	Contexts spanning multiple settings if they address paediatric community healthcare delivery	Settings outside healthcare (i.e., education)
		Universal health service
		Prevention programmes

Confidential: For Review Only

Information sources

The search included literature published from 1982 when SDM was first mentioned in scientific literature [9]. Only articles published in English were included due to the time and cost of transcription. A three-step search strategy was followed [18]. An initial search of Medline and CINAHL identified text words and index terms to develop a full search strategy (Table 2). This search strategy was reviewed by a librarian using the Peer Review of Electronic Search Strategies (PRESS) 2015 guideline [22] and was used to search databases including Medline, CINAHL, EMBASE, AMED,

PsycINFO, PubMed, Cochrane Database of Systematic Reviews and sources of grey literature including Open Grey, NICE guidelines, and CanChild website. The reference lists of included sources were screened for additional sources. The final search was completed on the 26th May 2022.

Table 2 Text words, index terms and subject heading identified for full search

Key concept	Text words / Index terms / MeSH terms (Medical Subject Headings) – combined using Boolean operators AND/OR
**Children**	child; child, preschool; adolescent; infant, extremely premature; infant; infant, newborn; paediatric
**Medical complexity**	medical complexity; special health care needs; disabilities; assistive technology; disabled children; developmental delay; chronic disease/th [therapy]; nervous system diseases/th [therapy]; medical fragility
**Shared decision-making**	parental decision-making; shared decision-making; parent perspective; decision making; patient participation; family-centred care; patient-centred care; professional-family relationship; parental discretion; bioethical issues;

Selection of evidence

Identified sources were uploaded to Covidence systematic review software and duplicates removed. Titles and abstracts were screened against criteria by (anon) and 33% was screened by (anon). Full texts of potentially relevant sources were assessed against inclusion criteria by (anon) and 33% by (anon). Reasons for exclusion of sources at full text were recorded. Disagreements between the reviewers were resolved through discussion and a third reviewer if needed (anon). The result of the search is outlined in a PRISMA flow chart (Figure 1).

Data charting process

The JBI source of evidence template [18] was modified for extraction of details about the author, publication year, country, participants, aim, context, study methods and findings relevant to the review questions. (Anon) completed data extraction and 10% were checked for consistency by (anon). Discrepancy in extraction were resolved through discussion. The extraction tool was iterative and was updated as the researchers became more familiar with the evidence.

Synthesis of results

Data was analysed by quantifying text, conducting basic qualitative content analysis and frequency counts [23]. Barriers and facilitators were ordered according to themes and mapped onto the ecological model of health behaviour (EMHB) [23,24]. The EMHB emphasizes the multiple layers of influence on healthcare behaviour and can guide the development of interventions by ensuring consideration of all factors impacting implementation [24]. Four ecological levels were used, 1) individual level including factors related to the child's needs, 2) family level relating to knowledge, attitudes, and skills of parents, 3) interpersonal level focused on interactions between HCPs and parents, and 4) organisational level considering institutional and HCP practices.

Confidential: For Review Only

Patient and public involvement statement

No patients were involved in conducting this scoping review.

RESULTS

Description of included sources

Thirty articles were included in this review , 18 were primary studies, eleven used qualitative [4,25–34] (Table 3), five quantitative [3,35–38] and two mixed methods [39,40] (Table 4). Eight articles were theoretical or opinion pieces [41–48] (Table 5) and four literature reviews [49–52] (Table 6). Twelve of the primary studies included parent participants, one included HCPs and five included parents and HCPs. Seventeen sources originated in the USA, seven in Canada, four in the UK, two in the Netherlands and one in South Korea. Research interest in SDM for CMC is increasing with 23 articles published in the last ten years of which 15 were published in the last five years.

Table 3 Description of sources – Qualitative studies (ordered from earliest publication date)

Lead author	Title	Country	Date	Aim
**Brotherson, M. [25]**	Quality of life issues for families who make the decision to use a feeding tube for their child with disabilities	United States	1995	To explore the issues families face in deciding whether to place a feeding tube.
**Katz, L. [26]**	A cultural interpretation of early intervention teams and the IFSP: parent and professional perceptions of roles and responsibilities.	United States	1995	To understand how members of an early intervention team involve families in developing of Individual Family Service Plans.
**Blue-Banning, M. [27]**	Dimensions of family and professional partnerships: Constructive guidelines for collaboration	United States	2004	To identify indicators of professional behaviour indicative of collaborative partnerships.
**Brotherton, A. [28,29]**	Mothers' process of decision making for gastrostomy placement	UK	2012	To explore mothers' constructions of decision making in gastrostomy feeding.
**Stille, C.J. [29]**	Parent Partnerships in Communication and Decision Making About Subspecialty Referrals for Children with Special Needs	Canada	2013	To describe factors influencing parent-clinician partnerships in SDM when children with special health care needs are referred to subspecialists.
**Zaal-Schuller, I.H. [30]**	How parents and physicians experience end-of-life decision-making for children with profound intellectual and multiple disabilities.	The Netherlands	2016	To compare experiences of parents and physicians involved in the end-of-life decision process.
**Buchanan, F. [31]**	What makes difficult decisions so difficult?: An activity theory analysis of decision making for physicians treating children with medical complexity.	Canada	2020	To first understand the complexity of the activity of decision-making.
**Lin, J.L. [32]**	Parent Perspectives in Shared Decision-Making for Children with Medical Complexity.	United States	2020	To identify components of SDM unique to the care of CMC from the perspective of parents.
**Jabre, N.A. [33]**	Parent perspectives on facilitating decision-making around pediatric home ventilation.	United States	2021	To understand parent perspectives about how clinicians can better facilitate decision-making around home ventilation.
**Reeder, J. [34]**	Becoming an empowered parent. How do parents successfully take up their role as a	UK	2021	To explore the important themes of dis/empowerment and the

Confidential: For Review Only

	collaborative partner in their child's specialist care?			influence of the therapeutic relationship.
**Buchanan , F [4]**	Decision-making for Parents of Children With Medical Complexities: Activity Theory Analysis.	Canada	2022	To explore decision-making of parents of CMC as an activity within the context of a process shared between clinician and parent.

Table 4 Description of sources – Quantitative and mixed methods studies (ordered from earliest publication date)

Lead author	Title	Country	Date	Aim
**Guerriere, D.N. [39]**	Mothers' decisions about gastrostomy tube insertion in children: factors contributing to uncertainty	Canada	2003	To explore mothers' perceptions of decision uncertainty.
**Denboba, D. [35]**	Achieving family and provider partnerships for children with special health care needs.	United States	2006	To assess whether families feel like partners in decision-making by their doctors.
**Pickering, D. [40]**	Disabled children's services: how do we measure family-centred care?	UK	2010	To evaluate staff and parental views of family-centred care in organizations providing services to young disabled children in Wales.
**Smalley, L.P. [36]**	Family perceptions of shared decision-making with health care providers: results of the National Survey of Children With Special Health Care Needs, 2009-2010.	United States	2014	To use data from a national survey to determine families' perceptions of SDM and determine the socio-demographic correlates.
**Lin, J.L. [3]**	Shared Decision Making among Children with Medical Complexity: Results from a Population-Based Survey.	Canada	2018	To compare the rates of SDM reported by parents of CMC with the rates of SDM reported by parents of non-complex children with special health care needs.
**Jolles, M.P. [38]**	Shared decision-making and parental experiences with health services to meet their child's special health care needs: Racial and ethnic disparities.	United States	2018	To test the relationship between SDM and parental report of frustration with efforts to get services for their child and to assess SDM's influence on minority parents' service experiences.
**An, M. [37]**	Effects of a Collaborative Intervention Process on Parent-Therapist Interaction: A Randomized Controlled Trial.	South Korea	2019	To determine whether collaborative intervention impacted interactions between parents of children with physical disabilities and physical therapists.

Table 5 Description of sources – Reviews (ordered from earliest publication date)

Lead author	Title	Country	Date	Aim
**Kruijsen-Terpstra, A.J.A [49]**	Parents' experiences with physical and occupational therapy for their young child with cerebral palsy: a mixed studies review.	The Netherlands	2013	To review literature on the experiences of parents of children with cerebral palsy with the physical and/or occupational therapy of their child.
**Popejoy, E. [50]**	Decision-making and future planning for children with life-limiting conditions: a qualitative	UK	2017	To synthesize findings from qualitative research about decision-

Confidential: For Review Only

	systematic review and thematic synthesis.			making and future planning for children with life-limiting conditions.
**Jonas, D. [51]**	Parental Decision-Making for Children With Medical Complexity: An Integrated Literature Review.	United States	2022	To consolidate existing literature on parental experience of medical decision making for CMC.
**LeGrow, K.R.N. [52]**	Relational Aspects of Parent and Home Health Care Provider Care Practices for Children With Complex Care Needs Receiving Health Care Services in the Home: A Narrative Review.	Canada	2022	To review literature on relational aspects of parent and home healthcare provider care practices for children with complex healthcare needs.

Table 6 Description of sources – Theoretical or opinion (ordered from earliest publication date)

Lead author	Title	Country	Date	Aim
**Bazyk, S. [41]**	Changes in Attitudes and Beliefs Regarding Parent Participation and Home Programs: An Update	United States	1989	To discuss traditional and current attitudes and practices regarding parent participation.
**Arvedson, J.C. [41]**	Ethical and legal challenges in feeding and swallowing intervention for infants and children	United States	2007	To outline current state of evidence-based decision making with feeding and swallowing.
**An, M. [43]**	Family-professional collaboration in pediatric rehabilitation: a practice model.	United States	2014	To describe a practice model of family-professional collaboration for paediatric rehabilitation.
**Austin, D. [44]**	Improving Partnerships to Make Family-Centered Care Work for Children with Special Health Care Needs.	United States	2014	Explaining the importance of partnership working from a parental perspective
**Adams, R.C. [45]**	Shared Decision-Making and Children with Disabilities: Pathways to Consensus.	United States	2017	To provide a basis for a systematic approach to implementation of SDM.
**Madrigal, V.N. [46]**	Supporting Family Decision-making for a Child Who Is Seriously Ill: Creating Synchrony and Connection	United States	2018	To discuss the process of supporting families facing chronic and serious illness during decision-making.
**Mahant, S. [47]**	Decision-making around gastrostomy tube feeding in children with neurologic impairment: Engaging effectively with families	Canada	2018	To review evidence and conceptual frameworks and provide recommendations to support decisions about gastrostomies.
**Lee, J.K. [48]**	Decision-Making for Children with Medical Complexity: The Role of the Primary Care Pediatrician.	United States	2020	Discussion of influences on decision-making from a paediatrician's perspective.

Definition of shared decision-making for children with medical complexity

Eighteen articles defined or described SDM (Figure 2). A collaborative approach and equal partnership between parents and HCPs were most frequently noted as key elements of SDM. Most other elements offered guidance on how to achieve this partnership. Three sources referenced the impact of uncertainty of prognosis and treatment outcomes for CMC on this collaborative process.

Confidential: For Review Only

Implementation of shared decision-making for children with medical complexity in community health services

Two qualitative [26,34] and two quantitative [3,36] studies reported on the implementation of SDM with CMC in community settings. The two qualitative studies were conducted 26 years apart (ethnographic study in 1995 [26] and interview-based study in 2021 [34]), in both parents perceived a power imbalance between them and HCPs with decision-making situated with HCPs. Two quantitative studies analysed the same dataset from a national survey in the USA [3,36] and found that although 85% of parents felt like partners with their child's doctor, there was a negative association with minority ethnic and low socio-economic status [36] and children with greater complexity [3]. This was attributed to multidisciplinary support needs, frequent hospital admissions, clinical uncertainty and social difficulties often experienced by parents of CMC.

Differences in shared decision-making in different communities

Eight of the eighteen primary studies reported on participant ethnicity but only three USA studies reported on differences in SDM among participants from a Black, Hispanic, and White background [35,36,38]. These studies found that families from minority ethnic backgrounds, those with lower educational backgrounds and lower income levels experienced less coordinated care and less SDM.

Barriers and facilitators to shared decision-making for children with medical complexity

Twenty-eight articles mentioned at least one barrier or facilitator to SDM. The most cited barriers related to clinical uncertainty of CMC, power imbalance between parents and HCPs and the lack of continuity of care (Table 7).

Table 7 Barriers to SDM for CMC mapped to the ecological model

**INDIVIDUAL (CHILD) LEVEL – theme: uncertainty**	
Uncertainty about diagnosis or clinical management options	[3,28,29,31,32,36,45,47,48,50]
Lack of evidence and uncertain illness trajectories	[3,25,31,32,45,47,50]
Limited or conflicting information	[25,29,30,39]
Uncertainty about child's comfort and quality if life	[36,50]
**FAMILY LEVEL**	
Language barriers	[29,36,38,40,45]
Poor general and health literacy	[25,28,45,50]
Lack of parental understanding of child's diagnosis and prognosis	[29,40,45]
Lack of trust in HCPs	[27,34,50]
Parents not feeling heard	[34,44,50]
Poverty, Black or minority ethnic background	[35,36]
Parental physical and emotional exhaustion and strong emotions	[32,50]
**INTERPERSONAL LEVEL – theme: power imbalance**	
Hierarchy and power imbalance, coercive conversations by HCP, failure to explain options fully or withholding information and labelling parents as non-compliant if they disagree with HCPs	[3,4,26,28–30,32,34,36,44,45,48,50]
Using medical jargon and providing too detailed information	[4,27,33,40,43,45,51]
HCPs not valuing parental opinion and experience	[30,32,34,45]
**ORGANISATIONAL LEVEL – theme: lack of continuity of care**	
Involvement of multiple subspecialities, lack of continuity of care	[3,4,32,40,44,45,48,50]

Confidential: For Review Only

Healthcare systems that dictate treatment options	[4,31,32,50]
Lack of time in consultations	[3,43,45]
Lack of professional understanding and valuing of SDM	[31,45]
Limited access to the right person to answer questions	[4,50]

The most cited facilitators to SDM for CMC included sharing of accessible, adequate, accurate and balanced information about all treatment options including knowing about uncertainty. Several facilitators related to the way HCPs viewed and engaged parents as active team members, service accessibility and attributes relating to the family (Table 8).

Table 8 Facilitators to SDM for CMC

**INDIVIDUAL (CHILD) LEVEL**	
Valuing the personhood of the child	[27,33,51]
**FAMILY LEVEL**	
Parental comfort with decision-making	[28,32,38,50,52]
Knowledge of the healthcare system	[4,50,51]
Parental educational level	[31,50]
Parental understanding of the child's diagnosis and prognosis	[34,50]
**INTERPERSONAL LEVEL**	
Mutual trust and respect	[4,27,29,35,46,48,49]
Actively empowering families to express their opinions, fears and hopes	[27,28,40,46,47,49]
Reciprocal good Interpersonal skills	[27,30,31,40,50]
Reciprocal active listening	[27,34,35,40,46]
Regarding parents as experts on their child	[29,40,49–51]
Having shared goals	[27,44,46,48]
Sensitivity to cultural differences	[27,33,40,50]
Showing dedication to the family	[27,33,51]
Professional awareness of parental decision-making preferences	[46,47]
Respecting family decisions	[26,31]
**ORGANISATIONAL LEVEL – theme: information and access**	
Having accessible, sufficient, accurate and balanced information about all treatment options including knowing about uncertainty	[4,25–27,30,32,33,46,47,51]
Access to peer-to-peer support	[25,29,33,36,38,47,50]
Having sufficient time to consider information and knowing the time-horizon for decision-making	[30,32,33,47,50,51]
Access to information from non-professional sources such as social media	[31,32,39,47,50]
Including parents as members of the team	[27,36,40]
Continuity of care	[33,40,51]
Access to interpreters if needed	[29,40]
Access to the right healthcare professionals to answer questions	[4,29]
HCP seeking advice from the wider team in the face of uncertainty	[31,51]
Having access to written information	[29,30]

Confidential: For Review Only

Research recommendations

Nineteen articles concluded with research recommendations, most related to discovering how to involve families and develop collaborative relationships, particularly families from diverse backgrounds (Table 9).

Table 9 Research recommendations

Discover how to involve families and develop collaborative relationships in SDM	[26,27,30,31,34,49,50,52]
Explore SDM within diverse communities, including diversity in family structure, culture and ethnicity, education level and healthcare setting	[30,32,36–38,49,51,52]
Explore family and healthcare professional's beliefs, perspectives, and experiences of SDM	[30–32,43,51,52]
Develop guidelines for SDM	[27,36,46,50]
Evaluate the effect of professional training on SDM	[27,34,37]
Evaluate proposed models of SDM	[36,43]
Develop outcomes measures for SDM	[45]
Develop support technologies for SDM	[45]
Investigate information needs for effective SDM	[25]

DISCUSSION

This scoping review explored the landscape of SDM for CMC in community health services. SDM is important in this population due to the complex long-term nature of their health conditions and high healthcare use. Like previous reviews [6,10], this review found no unifying definition for SDM in the literature. All sources highlighted the importance of SDM; however, few studies explored the effectiveness of SDM for CMC, especially in community health settings. This might in part be due to the varying nature of service delivery models in different countries The lack of implementation research is a shortcoming in paediatric research generally [10] with evidence mainly pertaining to adult care [5,17]. Limited research exists about SDM for CMC who are from a minority ethnic or disadvantaged background. Three studies showed poorer implementation in Black and Hispanic communities in the USA [35,36,38]. This is congruent with evidence from a systematic review that included studies from 15 countries, showing that adults from minority ethnic or disadvantaged backgrounds experience more barriers to SDM [53]. Research shows that SDM interventions can significantly improve the outcomes for disadvantaged adult patients, including increased knowledge and participation in decision-making [54]. It is therefore not surprising that exploring SDM in diverse communities was one of the most cited research recommendations.

Many influencing factors could either be barriers or facilitators to SDM depending on the context [5,7,53]. Most factors identified in this review are situated in the organisational level where HCPs can influence change. This include organisational and personal understanding of and commitment to SDM, interpersonal skills that build trust and respect, active listening, cultural sensitivity, empowerment of families to be active team members and to share their values, opinions and fears, continuity of care, access to the right HCPs and enough time in consultations. Previous studies found that the top patient-reported barriers to SDM include disorganised healthcare systems and the quality of interactions with HCPs [53], whereas the main barriers identified by HCPs are lack of time and motivation to pursue SDM and a perception that patients do not want to engage in decision-making [5,7]. There is however an awareness amongst HCPs that good communication and coordination of care can improve SDM [2]. To improve implementation of SDM it should be viewed as a culture within organisations and a way of interacting with every service-user rather than another clinical tool [2,10].

Another significant patient-identified barrier is hierarchy and power-imbalance still prevalent in traditional approaches, where HCPs are the main decision-makers [8]. Even when espousing SDM, HCPs often provide biased information in order to achieve a specific decisional outcome for instance

Confidential: For Review Only

by only providing information about their preferred treatment option [7]. This review found that HCPs often fail to explain all available options, withhold information, and use jargon. In some instances, HCPs provide too much and too detailed information, also negatively impacting the decision-making process. Effective information exchange is an important step in the decision-making process [55] and can either decrease or increase the power-balance in the relationship.

This power-imbalance is compounded by clinical information being held by HCPs who can influence the accuracy, clarity, tailoring and sharing of that information [7,13,53]. This review identified accessible, adequate, accurate and balanced information as one of the most significant contributors to successful SDM and conversely, the lack of evidence and information as a barrier to SDM. Providing information that outlines options, risks and uncertainties can improve SDM [7,8] and if presented in an accessible and culturally sensitive format, can help overcome language and socio-economic barriers such as poor literacy [53].

A complicating factor in SDM for CMC is however the lack of clinical and empirical evidence and information due to the unique illness trajectory of CMC, leading to high levels of uncertainty. The presence of uncertainty was the most striking barrier to SDM for CMC found in this review and sets CMC apart from many other patient populations. A high level of uncertainty is not a typical feature of SDM for children [7] or adults [53] but has been cited in areas such as neonatal intensive care [15], paediatric end-of-life care [15,56] and dementia end-of-life care [55]. The similarities with these clinical areas underscore the high-stress nature of decision-making for CMC [13,14].

This review highlights the need for further research to increase the evidence-base relating to diagnosis, prognosis, and treatment options for CMC and to address the implementation of SDM for CMC, specifically focussing on families from diverse backgrounds who often experience less SDM.

Strengths and limitations

This review contributes to the limited evidence-base concerning SDM for CMC and highlights themes around uncertainty, power-imbalance, and information sharing on implementation of SDM. The risk of missing sources due to the number of synonymous terms for SDM [6] and CMC [1] used in the literature was mitigated by developing a comprehensive list of search terms and conducting a systematic search using a range of databases. Results were strengthened by having second reviewers at all screening stages. Healthcare organisation and configuration vary across and within countries. It can range from mainly hospital-based services to services delivered in various hospital and community settings, services can be offered free at the point of contact or require payment by insurers or service-users. This variation could have impacted the identification of and comparability of studies. Most sources originated in the USA and Canada where barriers might be different to the UK and other parts of the world. The studies that included minority ethnic groups were conducted in the USA and do not represent the UK population. The review only included sources published in English which might have resulted in the exclusion of potentially valuable papers.

CONCLUSION

This scoping review revealed that uncertainty about diagnosis, prognosis, and treatment outcomes for CMC has a significant impact on SDM, in addition to barriers and facilitators identified in other paediatric and adult populations, highlighting the need to advance the clinical evidence base for this population. Furthermore, many factors impacting SDM fall within the organisational level where HCPs can influence change, including pursuing a power-balance and equal partnership, improving continuity of care, and improving information resources to meet the needs of parents of CMC, including those from diverse backgrounds. Focusing on these factors can potentially improve

Confidential: For Review Only

medical and developmental outcomes, quality of life of children and families and more effective use of healthcare resources. This review can be used to guide a research strategy in the field of SDM for CMC in community health services.

Contributors: SJ, ND and CS planned the study. SJ is the guarantor and took the lead in performing the scoping literature search and data analysis and writing the manuscript. KB, JO, and KS were second reviewers for source selection and extraction and reviewed the manuscript. ND and CS supervised the study and reviewed the manuscript. All authors critically reviewed the final draft of the manuscript.

Funding: SJ is an ICA Pre-doctoral Clinical Academic Fellow supported by Health Education England and the National Institute for Health Research, grant number: NIHR301944. The views expressed in this publication are those of the authors and not necessarily those of the NHS, the National Institute for Health Research or the Department of Health and Social Care.

Competing interests: None

Patient consent for publication: Not applicable

Data availability statement: Not applicable

References

1 Cohen E, Kuo DZ, Agrawal R, *et al.* Children with medical complexity: An emerging population for clinical and research initiatives. Pediatrics. 2011;**127**:529–38. doi:10.1542/peds.2010-0910

2 Birchley G, Thomas-Unsworth S, Mellor C, *et al.* Factors affecting decision-making in children with complex care needs: a consensus approach to develop best practice in a UK children's hospital. *BMJ Paediatr Open* 2022;**6**. doi:10.1136/bmjpo-2022-001589

3 Lin JL, Cohen E, Sanders LM. Shared Decision Making among Children with Medical Complexity: Results from a Population-Based Survey. *Journal of Pediatrics* 2018;**192**:216–22. doi:http://dx.doi.org/10.1016/j.jpeds.2017.09.001

4 Buchanan F, Lai C, Cohen E, *et al.* Decision-making for Parents of Children With Medical Complexities: Activity Theory Analysis. *J Particip Med* 2022;**14**:e31699. doi:https://dx.doi.org/10.2196/31699

5 Gravel K, Légaré F, Graham ID. Barriers and facilitators to implementing shared decision-making in clinical practice: A systematic review of health professionals' perceptions. Implementation Science. 2006;**1**. doi:10.1186/1748-5908-1-16

Confidential: For Review Only

6 Park ES, Cho IY. Shared decision-making in the paediatric field: a literature review and concept analysis. Scand J Caring Sci. 2018;**32**:478–89. doi:10.1111/scs.12496

7 Boland L, Graham ID, Légaré F, *et al.* Barriers and facilitators of pediatric shared decision-making: A systematic review. Implementation Science. 2019;**14**. doi:10.1186/s13012-018-0851-5

8 Coulter Angela, Collins A, King's Fund Centre (London). *Making shared decision-making a reality: no decision about me, without me*. King's Fund 2011. https://www.kingsfund.org.uk/publications/making-shared-decision-making-reality (accessed 16 Oct 2022).

9 Mulley AG, Trimble Chris, Elwyn Glyn. *Patients' preferences matter: stop the silent misdiagnosis*. King's Fund 2012. https://www.kingsfund.org.uk/publications/patients-preferences-matter (accessed 10 Aug 2022).

10 Wyatt KD, List B, Brinkman WB, *et al.* Shared Decision Making in Pediatrics: A Systematic Review and Meta-analysis. *Acad Pediatr* 2015;**15**:573–83. doi:10.1016/j.acap.2015.03.011

11 Vemuri S, Hynson J, Williams K, *et al.* Decision-making approaches for children with life-limiting conditions: results from a qualitative phenomenological study. *BMC Med Ethics* 2022;**23**. doi:10.1186/s12910-022-00788-7

12 Fiks AG, Hughes CC, Gafen A, *et al.* Contrasting parents' and pediatricians' perspectives on shared decision-making in ADHD. *Pediatrics* 2011;**127**. doi:10.1542/peds.2010-1510

13 Dias BC, Marcon SS, Reis P dos, *et al.* Family dynamics and social network of families of children with special needs for complex/continuous cares. *Rev Gaucha Enferm* 2020;**41**:e20190178. doi:10.1590/1983-1447.2020.20190178

14 Woodgate RL, Edwards M, Ripat JD, *et al.* Intense parenting: A qualitative study detailing the experiences of parenting children with complex care needs. *BMC Pediatr* 2015;**15**. doi:10.1186/s12887-015-0514-5

15 Krick JA, Hogue JS, Reese TR, *et al.* Uncertainty: An Uncomfortable companion to decision-making for infants. *Pediatrics* 2020;**146**:S14–7. doi:10.1542/peds.2020-0818E

16 Jacobs S, Davies N, Smith CH. Shared decision-making for children with medical complexity in community health services – scoping review protocol. *Open Science Framework* Published Online First: 2022. doi:DOI 10.17605/OSF.IO/VJ2KB

17 Joseph-Williams N, Lloyd A, Edwards A, *et al.* Implementing shared decision making in the NHS: lessons from the MAGIC programme. *BMJ* 2017;**357**:j1744. doi:10.1136/bmj.j1744

18 Peters MDJ, Marnie C, Tricco AC, *et al.* Updated methodological guidance for the conduct of scoping reviews. *JBI Evid Synth* 2020;**18**:2119–26. doi:10.11124/JBIES-20-00167

19 Peters MDJ, Godfrey C, Mcinerney P, *et al.* Best practice guidance and reporting items for the development of scoping review protocols. *JBI Evid Synth* 2022;**20**:1–19. doi:10.11124/JBIES-21-00242

20 Jordan PZ. JBI Manual for Evidence Synthesis. 2020. doi:10.46658/jbimes-20-01

21 Tricco AC, Lillie E, Zarin W, *et al.* PRISMA extension for scoping reviews (PRISMA-ScR): Checklist and explanation. Ann Intern Med. 2018;**169**:467–73. doi:10.7326/M18-0850

Confidential: For Review Only

22 McGowan J, Sampson M, Salzwedel DM, *et al.* PRESS Peer Review of Electronic Search Strategies: 2015 Guideline Statement. *J Clin Epidemiol* 2016;**75**:40–6. doi:10.1016/j.jclinepi.2016.01.021

23 Pollock D, Peters MDJ, Khalil H, *et al.* Recommendations for the extraction, analysis, and presentation of results in scoping reviews. JBI Evid Synth. [preprint] October 14, 2022 doi: 10.11124/JBIES-22-00123. *Ashrita Saran* 2022;**10**:18. doi:10.11124/JBIES-22-00123

24 Sallis JF, Owen N, Fisher E. Ecological Models of Health Behavior. In: Glanz K, Rimer BK, Viswanath K, eds. *Health Behavior and Health Education: Theory, Research and Practice*. Jossey-Bass 2008. 465–86.https://www.researchgate.net/publication/43508113 (accessed 28 Nov 2022).

25 Brotherson MJ, Oakland MJ, Secrist-Mertz C, *et al.* Quality of Life Issues for Families Who Make the Decision to Use a Feeding Tube for Their Child With Disabilities. *Journal of the Association for Persons with Severe Handicaps* 1995;**20**:202–12. doi:doi.org/10.1177/154079699502000305

26 Katz L, Scarpati S. A Cultural Interpretation of Early Intervention Teams and the IFSP: Parent and Professional Perceptions of Roles and Responsibilities. *Infant-Toddler Intervention: The Transdisciplinary Journal* 1995;**5**:177–91.https://www.researchgate.net/publication/234649683

27 Blue-Banning M, Summers JA, Frankland HC, *et al.* Dimensions of family and professional partnerships: Constructive guidelines for collaboration. *Except Child* 2004;**70**:167–84. doi:10.1177/001440290407000203

28 Brotherton A, Abbott J. Mothers' process of decision making for gastrostomy placement. *Qual Health Res* 2012;**22**:587–94. doi:10.1177/1049732311423841

29 Stille CJ, Fischer SH, Nancy ;, *et al.* Parent Partnerships in Communication and Decision Making About Subspecialty Referrals for Children With Special Needs. *Acad Pediatr* 2013;**13**:122–32. doi:DOI: 10.1016/j.acap.2012.12.003

30 Zaal-Schuller, Willems DL, Ewals FVPM, *et al.* How parents and physicians experience end-of-life decision-making for children with profound intellectual and multiple disabilities. *Res Dev Disabil* 2016;**59**:283–93. doi:https://dx.doi.org/10.1016/j.ridd.2016.09.012

31 Buchanan F, Cohen E, Milo-Manson G, *et al.* What makes difficult decisions so difficult?: An activity theory analysis of decision making for physicians treating children with medical complexity. *Patient Educ Couns* 2020;**103**:2260–8. doi:10.1016/j.pec.2020.04.027

32 Lin, Clark CL, Halpern-Felsher B, *et al.* Parent Perspectives in Shared Decision-Making for Children With Medical Complexity. *Acad Pediatr* 2020;**20**:1101–8. doi:10.1016/j.acap.2020.06.008

33 Jabre NA, Raisanen JC, Shipman KJ, *et al.* Parent perspectives on facilitating decision-making around pediatric home ventilation. *Pediatr Pulmonol* 2022;**57**:567–75. doi:10.1002/ppul.25749

34 Reeder J, Morris J. Becoming an empowered parent. How do parents successfully take up their role as a collaborative partner in their child's specialist care? *Journal of Child Health Care* 2021;**25**:110–25. doi:10.1177/1367493520910832

Confidential: For Review Only

35 Denboba D, McPherson MG, Kenney MK, *et al.* Achieving family and provider partnerships for children with special health care needs. *Pediatrics* 2006;**118**:1607–15. doi:DOI: 10.1542/peds.2006-0383

36 Smalley, Kenney M, Denboba D, *et al.* Family Perceptions of Shared Decision-Making with Health Care Providers: Results of the National Survey of Children with Special Health Care Needs, 2009-2010. *Matern Child Health J* 2014;**18**:1316–27. doi:10.1007/s10995-013-1365-z

37 An M, Palisano RJ, Yi CH, *et al.* Effects of a Collaborative Intervention Process on Parent-Therapist Interaction: a Randomized Controlled Trial. *Phys Occup Ther Pediatr* 2019;**39**:259-275. doi:10.1080/01942638.2018.1496965

38 Jolles MP, Lee PJ, Javier JR. Shared decision-making and parental experiences with health services to meet their child's special health care needs: Racial and ethnic disparities. *Patient Educ Couns* 2018;**101**:1753–60. doi:10.1016/j.pec.2018.05.022

39 Guerriere DN, Mckeever P, Llewellyn-Thomas H, *et al.* Mothers' decisions about gastrostomy tube insertion in children: factors contributing to uncertainty. *Dev Med Child Neurol* 2003;**45**:470–6. doi:10.1017/S0012162203000872

40 Pickering D, Busse M. Disabled children's services: How do we measure family-centred care? *Journal of Child Health Care* 2010;**14**:200–7. doi:10.1177/1367493509360207

41 Arvedson JC, Lefton-Greif MA. Ethical and legal challenges in feeding and swallowing intervention for infants and children. *Semin Speech Lang* 2007;**28**:232–8. doi:10.1055/s-2007-984729

42 Bazyk S. Changes in Attitudes and Beliefs Regarding Parent Participation and Home Programs: An Update. *American Journal of Occupational Therapy* 1989;**43**:723–8. doi:10.5014/ajot.43.11.723

43 An M, Palisano RJ. Family-professional collaboration in pediatric rehabilitation: a practice model. *Disabil Rehabil* 2014;**36**:434–40. doi:10.3109/09638288.2013.797510

44 Austin D. Improving Partnerships to Make Family-Centered Care Work for Children with Special Health Care Needs. Science of Caring. 2014;:1.https://scienceofcaring.ucsf.edu/community/improving-partnerships-make-family-centered-care-work-children-special-health-care-needs (accessed 2 Mar 2022).

45 Adams, Levy SE. Shared Decision-Making and Children With Disabilities: Pathways to Consensus. *Pediatrics* 2017;**139**:1–9.https://doi.org/10.1542/peds.2017-0956

46 Madrigal VN, Patterson Kelly K, Washington G. Supporting Family Decision-making for a Child Who Is Seriously Ill: Creating Synchrony and Connection. *Pediatrics* 2018;**142**:S170–7. doi:10.1542/peds.2018-0516H

47 Mahant S, Cohen E, Nelson KE, *et al.* Decision-making around gastrostomy tube feeding in children with neurologic impairment: Engaging effectively with families. *Paediatrics and Child Health (Canada)* 2018;**23**:209–13. doi:10.1093/pch/pxx193

48 Lee KJ, Hill DL, Feudtner C. Decision-making for children with medical complexity: The role of the primary care pediatrician. *Pediatr Ann* 2020;**49**:e473–7. doi:10.3928/19382359-20201013-01

Confidential: For Review Only

49 Kruijsen-Terpstra AJ, Ketelaar M, Boeije H, *et al.* Parents' experiences with physical and occupational therapy for their young child with cerebral palsy: a mixed studies review. *Child Care Health Dev* 2014;**40**:787–96. doi:http://dx.doi.org/10.1111/cch.12097

50 Popejoy E, Pollock K, Almack K, *et al.* Decision-making and future planning for children with life-limiting conditions: a qualitative systematic review and thematic synthesis. *Child Care Health Dev* 2017;**43**:627–44. doi:10.1111/cch.12461

51 Jonas, Scanlon C, Bogetz JF. Parental Decision-Making for Children With Medical Complexity: An Integrated Literature Review. *J Pain Symptom Manage* 2022;**63**. doi:10.1016/j.jpainsymman.2021.07.029

52 LeGrow, Cohen E, Espin S. Relational Aspects of Parent and Home Health Care Provider Care Practices for Children With Complex Care Needs Receiving Health Care Services in the Home: A Narrative Review. *Acad Pediatr* 2022;**22**:196–202. doi:10.1016/j.acap.2021.08.009

53 Joseph-Williams N, Elwyn G, Edwards A. Knowledge is not power for patients: A systematic review and thematic synthesis of patient-reported barriers and facilitators to shared decision making. Patient Educ Couns. 2014;**94**:291–309. doi:10.1016/j.pec.2013.10.031

54 Durand MA, Carpenter L, Dolan H, *et al.* Do interventions designed to support shared decision- making reduce health inequalities? A systematic review and meta-analysis. *PLoS One* 2014;**9**. doi:10.1371/journal.pone.0094670

55 Davies N, de Souza T, Rait G, *et al.* Developing an applied model for making decisions towards the end of life about care for someone with dementia. *PLoS One* 2021;**16**. doi:10.1371/journal.pone.0252464

56 Richards CA, Starks H, O'Connor MR, *et al.* Physicians Perceptions of Shared Decision-Making in Neonatal and Pediatric Critical Care. *American Journal of Hospice and Palliative Medicine* 2018;**35**:669–76. doi:10.1177/1049909117734843

Figures:

Figure 1 PRISMA flow chart

Figure 2 Elements of shared decision-making for children with medical complexity

Confidential: For Review Only

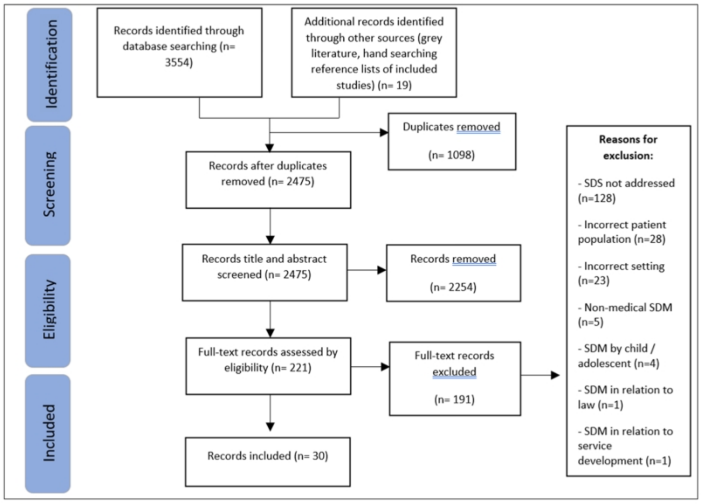

Figure 1 PRISMA flow chart

533x754mm (118 x 118 DPI)

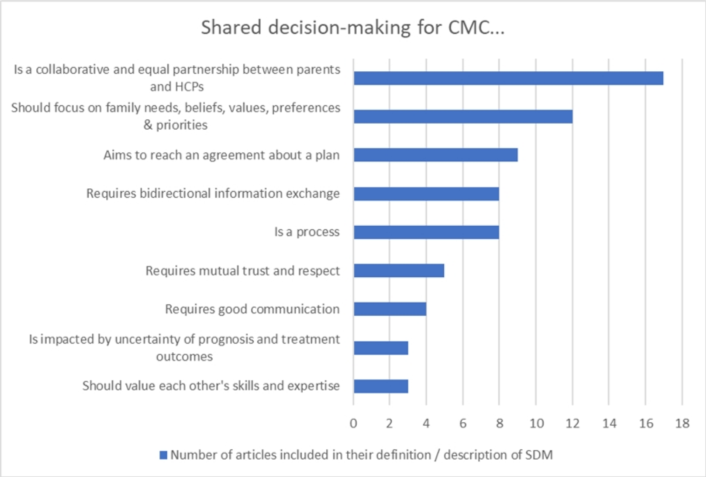

Figure 2 Elements of shared decision-making for children with medical complexity

533x754mm (118 x 118 DPI)
